# Controlled movement of ssDNA conjugated peptide through *Mycobacterium smegmatis* porin A (MspA) nanopore by a helicase motor for peptide sequencing application†

**DOI:** 10.1039/d1sc04342k

**Published:** 2021-11-12

**Authors:** Zhijie Chen, Zhenqin Wang, Yang Xu, Xiaochun Zhang, Boxue Tian, Jingwei Bai

**Affiliations:** School of Pharmaceutical Sciences, Tsinghua University 100084 Beijing China jingwbai@tsinghua.edu.cn

## Abstract

The lack of an efficient, low-cost sequencing method has long been a significant bottleneck in protein research and applications. In recent years, the nanopore platform has emerged as a fast and inexpensive method for single-molecule nucleic acid sequencing, but attempts to apply it to protein/peptide sequencing have resulted in limited success. Here we report a strategy to control peptide translocation through the MspA nanopore, which could serve as the first step toward strand peptide sequencing. By conjugating the target peptide to a helicase-regulated handle-ssDNA, we achieved a read length of up to 17 amino acids (aa) and demonstrated the feasibility of distinguishing between amino acid residues of different charges or between different phosphorylation sites. Further improvement of resolution may require engineering MspA-M2 to reduce its constriction zone's size and stretch the target peptide inside the nanopore to minimize random thermal motion. We believe that our method in this study can significantly accelerate the development and commercialization of nanopore-based peptide sequencing technologies.

## Abstract 2

A new technique for single molecular peptide sequencing is demonstrated by translocation of ssDNA-conjugated-peptide through MspA nanopore which is regulated by a DNA helicase motor.

## Graphical abstract

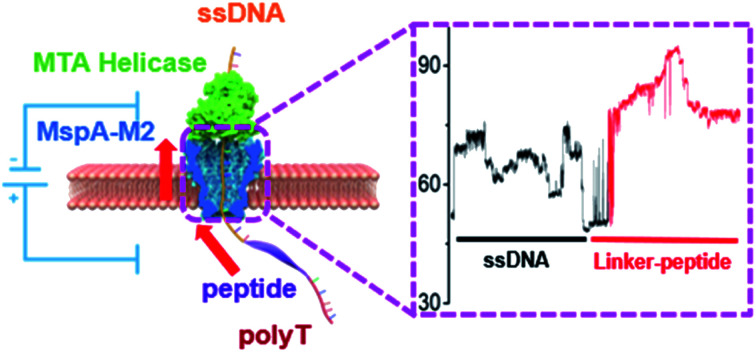

## Introduction

Protein sequencing is an indispensable tool for discovering new proteins and characterizing the post-translational modifications to elucidate their functional dynamics.^1,2^ Unfortunately, current methods, such as Edman degradation and mass spectrometry, are inefficient, complicated to execute, and expensive due to instrument costs.^3–6^ Protein fluorosequencing has the advantage of reading millions of oligo-peptides simultaneously, but its detection is limited to those amino acid residues amenable to fluorophore labeling.^7,8^ Recently, the nanopore technology has emerged as a highly sensitive and inexpensive DNA sequencing method,^9^ which prompted a flurry of research aimed to apply it to protein identification.^9–11^ In fact, several studies have already demonstrated the feasibility of protein unfolding and translocation through either aerolysin or solid-state nanopores.^12–15^ Nivala *et al.* utilized the ClpX unfoldase as a motor protein to regulate the speed of polypeptide translocation through the α-hemolysin pore.^16,17^ Despite the improvement, the strategy was unable to accurately identify individual amino acid residues due to the low spatial sensitivity of the α-hemolysin pore, as well as the overly fast and uneven translocation of the peptide strand.

Compared to well-studied nucleic acid motors, such as polymerases, helicases, translocases, motor proteins that bind to and transport polypeptides have not been extensively characterized.^18,19^ To address the poor availability of reliable peptide motor proteins, we hypothesized that nanopore-based peptide transport could be controlled by conjugating the peptide to a single-stranded DNA (ssDNA) handle under the regulation of a DNA helicase. In fact, similar strategies have previously been employed in the investigation of enzyme dynamics,^20^ direct microRNA sequencing, and epigenetic analysis.^21,22^ We selected MspA-M2 (D90N/D91N/D93N/D118R/E139K/D134R) as the model nanopore because its constriction is located at the very bottom of the channel, which could theoretically lead to better read length.^23,24^ We demonstrated that peptides up to 17 amino acid residues could be threaded through the constriction of MspA-M2. Based on the temporal pattern of the generated ionic current, we were able to differentiate point mutations based on their charges and locations in the backbone, as well as phosphorylation sites. Further improvement could be achieved by shrinking the constriction zone and stretching the peptide chain with an enzyme or applying osmotic flow.^25,26^ Combined, these strategies may provide a feasible pathway to efficient, low-cost peptide sequencing.

## Result and discussion

### Pulling ssDNA-peptide conjugates through MspA-M2 nanopore with a helicase motor

**Fig. 1:**
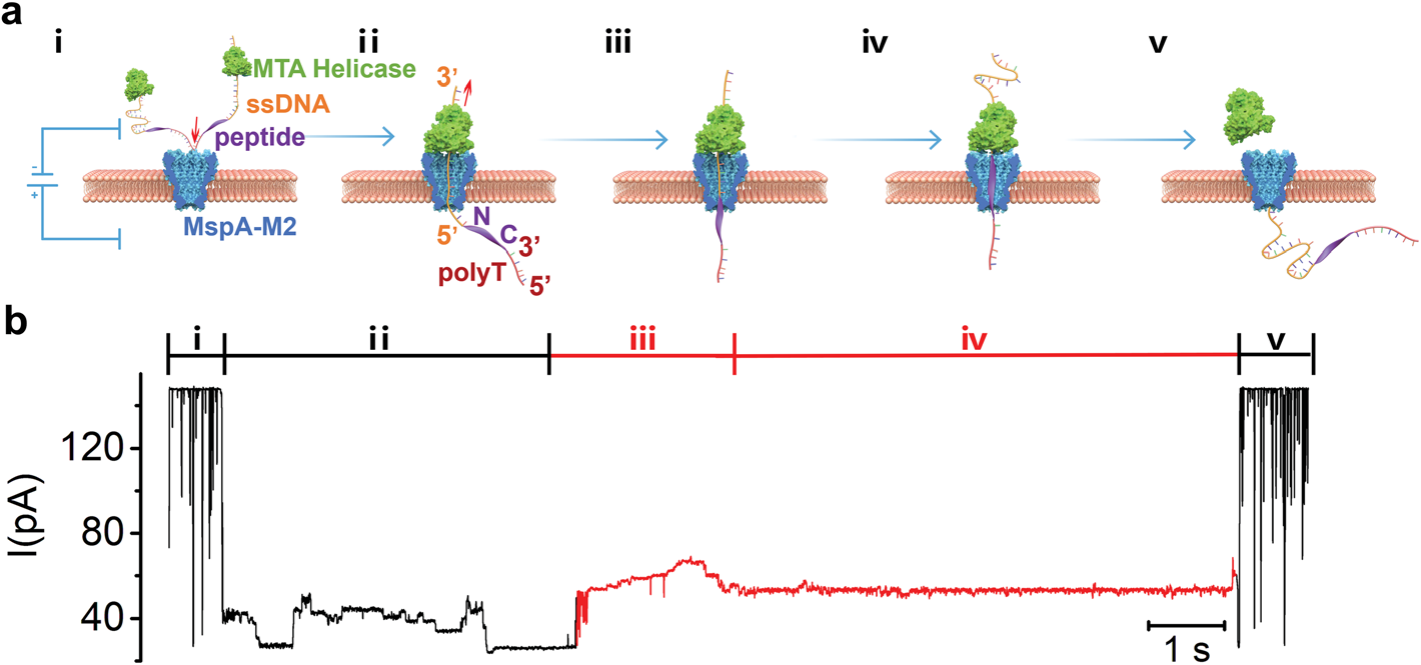
(a) Schematic view of peptide sequencing achieved by helicase-driven translocation of DNA-peptide conjugates through the MspA-M2 nanopore. Step i to V illustrate the different stages of sequencing event which can be reflected in the ionic current signal. The *cis* chamber solution containing free MTA helicase, DNA-peptide conjugates and helicase bond DNA-peptide conjugates. Under 180 mV applied potential, the negative charged DNA-peptide conjugates tend to enter the pore from the *cis* side to *trans* side (step i), and stopp if the bond helicase is stuck on the top rim of the nanopore protein. The sliding of the helicase on ssDNA in 3′-to-5′ direction drives the DNA-peptide conjugate to move upward against the electrical field. Therefore, it is the handle-ssDNA passed the MspA constriction first (step ii), and then is the peptide (step iii). The movement of the DNA-peptide conjugate stops when the helicase reaches the peptide region (step iv), resulting it detaching from the helicase and translocate back through nanopore under electrical field (step v). (b) The sequencing signal of the DNA-peptide conjugate with the changes of ionic current profile corresponding to the stages of the translocation marked in (a). The DNA-peptide conjugates is constructed in the form of 3′-ssDNA(89)-5′-N-peptide(23)-C-3′-polyT(30)-5′ with full sequence: 3′-CTACTACTTTTTTCTTTTTTCGATGCTGGACGTACTCTTACGCTAT CACTCTAGACTTTTTTTTTTTTTTTTTTTTTTTTTTTTTTTTT-5′-N – GGGGSGGGGSSGGGGSSGGSGGC-3′-TTTTTTTTTTTTTTTTTTTTTTTTTTTTTT-5′.

The principle of strand peptide sequencing is illustrated in Fig. 1a. First, the N-terminal of the target peptide is chemically conjugated *via* click reaction to the 5′-end of a handle-ssDNA (ssDNA) (Table S2†) which binds to a *Methane algae thermophilic* helicase (MTA-h), a member of the Hel308 family. Similar to the Hel308 Mbu and Tga helicases already in use for long-read DNA sequencing,^20^ MTA-h shows significant salinity tolerance and remains functional even at a salt concentration of 600 mM, making it well suited for strand sequencing (Fig. S1, S2†). In order to minimize the interference of any possible secondary structures, we tested a 23-aa flexible polypeptide chain with a C-terminal cysteine residue (GGGGSGGGGSSGGGGSSGGSGGC), which, however, is insensitive to the electrical field due to the charge neutrality of its amino acid residues. We improved the signal generation ability of the peptide by conjugating the C-terminal cysteine residue to a maleimide-modified 30 nucleotides poly-T lead-ssDNA (polyT) (Fig. S3†), resulted in a sandwiched polymer structure: 3′-ssDNA(89)-5′-N-peptide(23)-C-3′-polyT(30)-5’. Under an electrical field, the 5′ end of the polyT enters MspA-M2 from its *cis* side and moves through the channel until the bulky MTA-h blocks the top rim of the entrance (Fig. 1a, step i, ii). Once stuck, the inherent ability of MTA to slide along the ssDNA in the 3′-to-5′ direction will cause the conjugate strand to backtrack through the pore, beginning with the ssDNA segment and followed by the peptide (Fig. 1a, step ii, iii). When the strand passes through the nanopore constriction, it generates a blocking current whose amplitude pattern is governed largely by the biochemical nature of the sequence.^27,28^ Typically, the current profile begins with a long and relatively stable signal generated by the ssDNA, whereas the subsequent sudden amplitude surge, followed by fluctuations, can be attributed to the peptide translocation (Fig. 1b).

When the conjugate strand traversed through MspA-M2, the ssDNA handle threaded through the constriction first, which produced a step-like current that ended with a long and flat blocking current of around 27 pA (Fig. 1b, S4†). The blocking current then showed a sudden rise in amplitude to 53 pA, which we theorized could be attributed to the beginning of peptide translocation, as amino acids are smaller than nucleotides (Fig. 1b). The signal then continued to intensify, albeit at a significantly more moderate pace, to a peak value of about 69 pA, before attenuating to a flat trace of 54 pA, which likely signified the cessation of MTA movement at the ssDNA-peptide junction. Eventually, the current blocking profile could end with either an abrupt elevation to the open-pore current or decline further to the amplitude of the polyT lead sequence, depending on the peptide length. Instead of showing a reproducible, step-like pattern characteristic of a typical ssDNA signal,^28^ the blocking current generated by the peptide strand first showed a steep surge in amplitude, and then alternated between periods of relatively moderate rise and fall, interspersed occasionally by step-like, but unreproducible signals. We speculated that the largely charge-neutral peptide chain might not be fully stretched in the constriction zone, which prevented it from synchronizing with the pace of the helicase motor and thus resulted in continuous, rather than intermittent, strand movement. Additionally, although the length of the constriction of MspA-M2 is only 0.6 nm, which is on par with the average length of the interval between two consecutive nucleotides in a stretched DNA sequence, molecular dynamic studies have previously indicated that the neighbouring nucleotides could also occlude the nanopore passage due to thermal motion, resulting a k-mer length of at least 3 nucleic acid bases.^29–31^ Because the average peptide strand has a shorter unit distance and a more flexible backbone than ssDNA, a greater number of neighbouring amino acid residues can contribute to the blocking current simultaneously in nanopore protein sequencing compared to nucleotides in DNA sequencing. As a result, the increase in the k-mer length led to signal degeneration by smoothing what would have been step-like signatures of consecutive amino acids into a continuous slope.

### Determination of sequencing read length

**Fig. 2:**
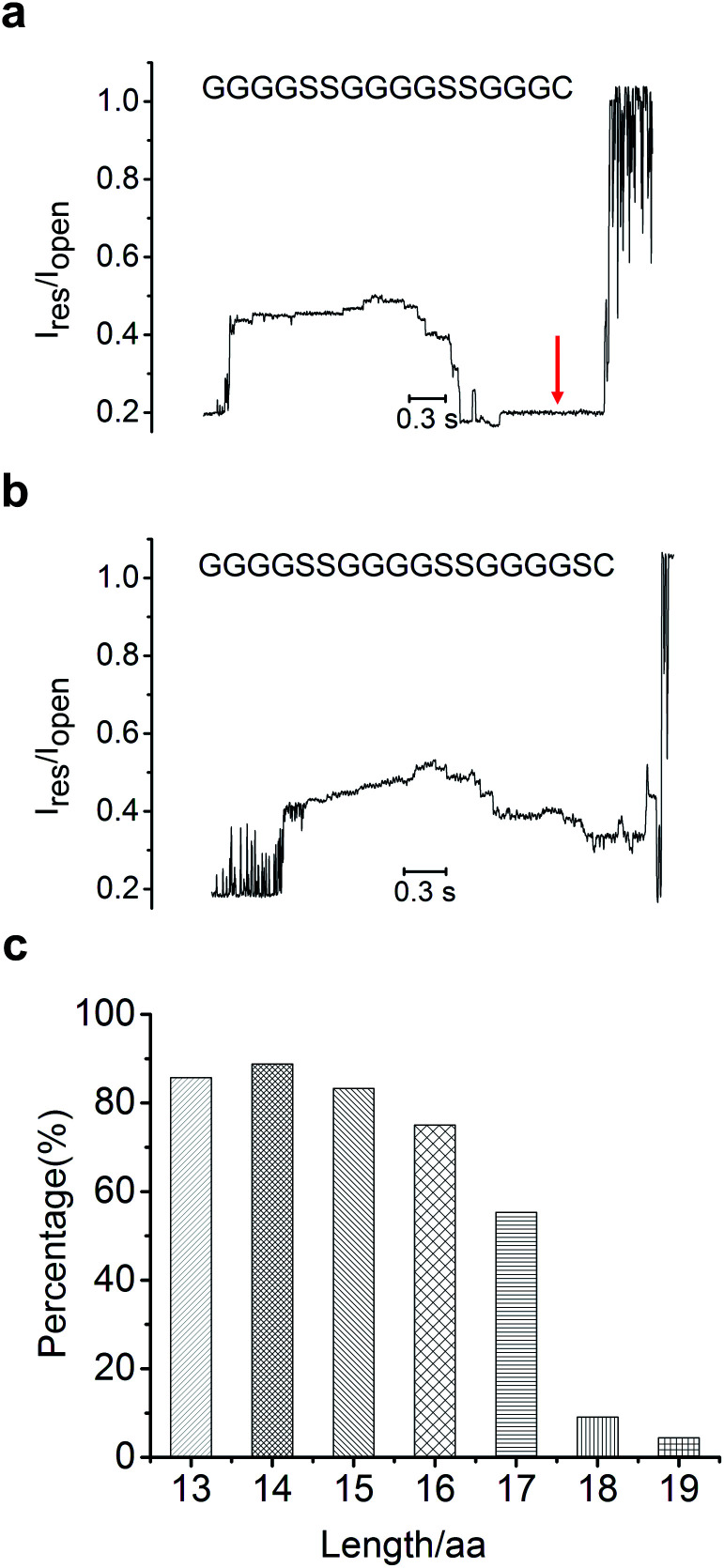
Ionic current signals of peptide length of 16 amino acids (a) and 18 amino acids (b); the red arrow pointed to the signal from the polyT sequence at the 5′ end of the sandwiched 3′-ssDNA-5′-N-peptide-C-3′-polyT-5′ conjugates. (c) Percentage of translocation events in which the polyT signal was detected for peptides of each length. The open-pore current is typically around 150 pA under the experimental conditions employed.

Since the MTA-h can only move along ssDNA and stop at the peptide conjugate site, this limits the sequencing read length, defined as the number of amino acid residues that can be pulled back through the constriction. Therefore, the read length is governed by the distance between the helicase and the constriction, which is limited to about 70 angstroms for MspA-M2, roughly equivalent to the combined length of 13 nucleotides or 17 amino acids (with the 1-nm long linker).^32^ To verify the read length of peptide sequencing, we synthesized a series of polypeptides in different lengths (Table S3†), and determined whether each full-length peptide could be completely led back through the constriction by monitoring the polyT induced flat current signature after the peptide signal(Fig. 2, S5†). To compare the signal profiles of different peptides, we measured the scaled ratio of the translocation-induced ionic current to the open-pore current (I_res_/I_open,_ ESI†), as shown in Fig. 2. Indeed, conjugate strands in which the peptide segment was shorter than 17 amino acids produced a polyT signal at about 0.19 I_open_ in 70% to 90% of the sequencing events (Fig. 2a, c). For the 17-aa peptide, the probability of observing the polyT signal in the blocking current profile dropped to 50% (Fig S5e, f†), and the translocation of polyT was rarely observed for peptides with 18 amino acids or more (Fig. 2b, c). These results indicated that our method could detect peptides with lengths up to 17 amino acids, consistent with the structural analysis of MspA-M2.^33^

**Fig. 3:**
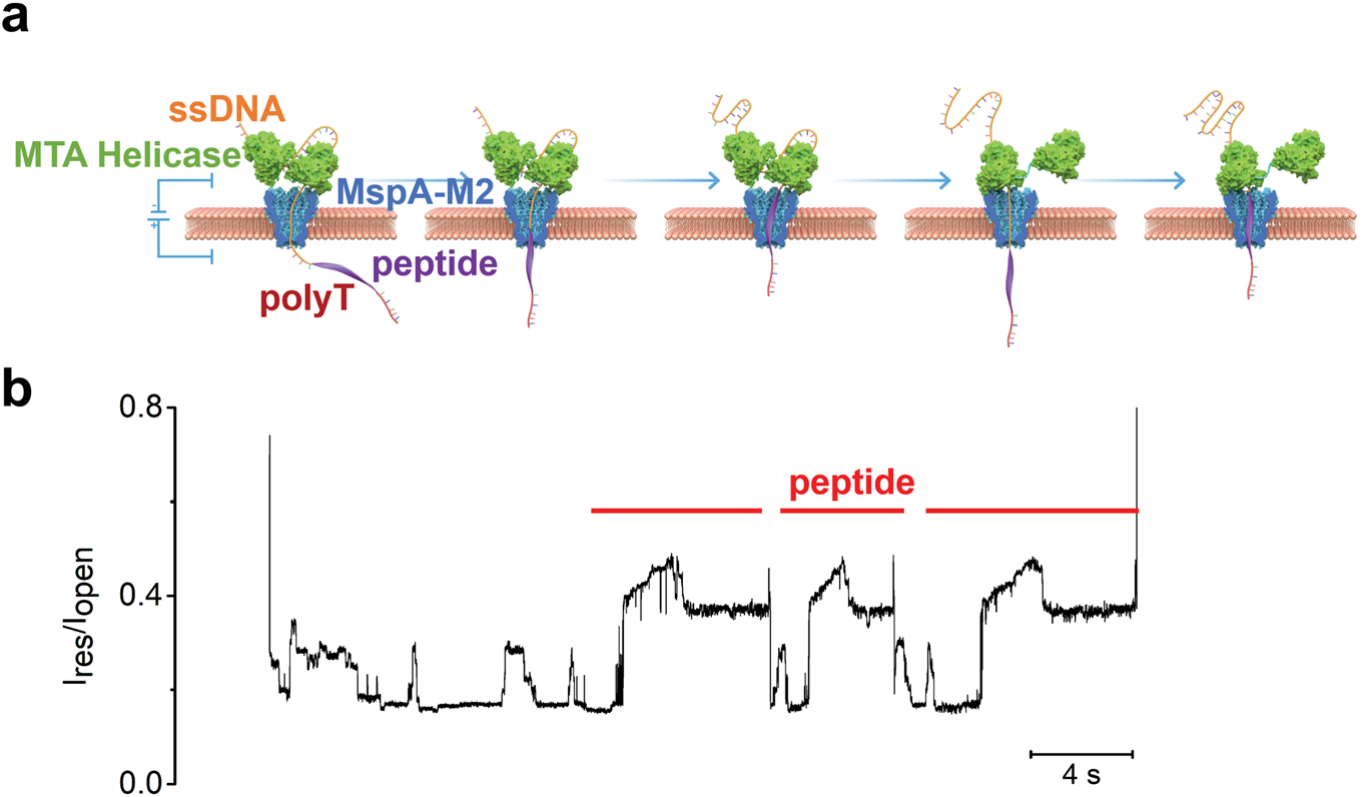
(a) Illustration of the mechanism for repeated sequencing with a dual-helicase construct. (b) The experimental result showing at least three repeated peptide translocation events; the sequencing signal of ssDNA (6-89T from Table S2†) can also be observed between two high current signatures induced by peptide sequence (GGGGFGGGGSSGGGGSSGGSGGC).

To further improve the read length, we envisaged a tandem arrangement of two MTA-h units to achieve coordinated ssDNA transport over a longer distance (Fig. 3a). Because the length of the ssDNA exceeds that of a single MspA-M2 channel, it will thread through the bottom MTA-h and enter the top helicase unit before the peptide conjugate site reaches the bottom MTA-h and causes it to cease the strand transport. At this point, the top MTA-h will continue to pull the sequence upward until it stops at the ssDNA-peptide junction, ultimately leading to increased sequencing length. Unfortunately, although our initial experiments showed that the dual-MTA-h construct had a greater binding affinity for ssDNA, suggesting that both helicase units could bind to the nucleic acid sequence (Fig. S2†), we did not observe an increase in read length. Interestingly instead, the blocking current profile showed two or more peptide translocation signals in a row, providing evidence that the same peptide was repeatedly sequenced (Fig. 3b). These observations combined prompted us to theorize that the strand dissociation from the bottom MTA-h at the ssDNA-peptide junction resulted in unregulated nanopore translocation; however, as the strand backtracked away from the MTA-h units, the dual-helicase system had a better chance to recapture the ssDNA and thus re-initiate the pulling process, leading to repeated sequencing of the same peptide (Fig. 3a). Of note, a similar strategy has been incorporated in the Single Molecule, Real-Time (SMRT) Sequencing platform to improve the accuracy of single-molecule DNA sequencing from about 90% to over 99%.^34^ Therefore, the dual-MTA-h system can potentially improve the accuracy of peptide sequencing by repeatedly reading the same strand and analysing the signal consensus.^35^

### Differentiation of amino acid residue

**Fig. 4:**
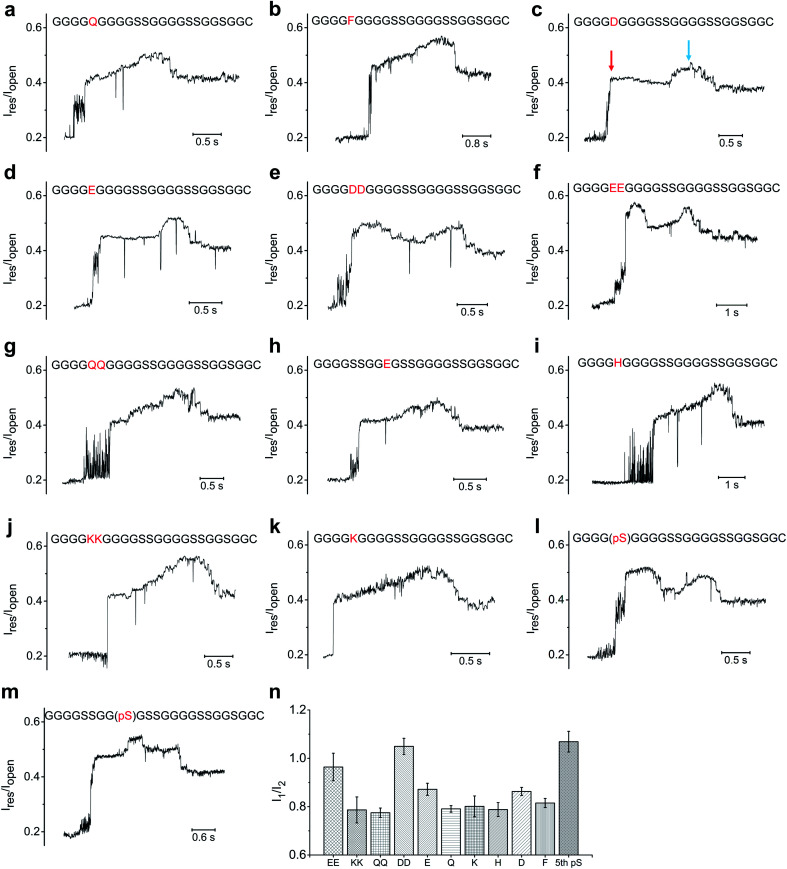
The ionic current profile of different mutants on polypeptide (GGGGSGGGGSSGGGGSSGGSGGC): (a) S_5_Q; (b) S_5_F; (c) S_5_D; (d) S_5_E; (e)S_5_DD; (f) S_5_EE; (g) S_5_QQ; (h) S_5_SS, G_9_E; (i) S_5_H; (j) S_5_KK; (k) S_5_k; (l) phosphorylation of S_5_; (m) S_5_SS, phosphorylation of G_9_S. (n) Comparison between I_1_, which is measured based on the amplitude level of the initial steep current level rising, and I_2_, which is the amplitude level of the main peak. The blue arrows point to the main peak; the red arrows point to the type of initial current rising (hump) induced by the negatively charged mutations or phosphorylation.

Based on above experimental setup, we then investigated whether we could distinguish between different types of amino acids. We first mutated the serine at the fifth position (designated as S_5_) from the N-terminus of the 23-aa polypeptide (GGGGSGGGGSSGGGGSSGGSGGC) to an array of neutral/hydrophobic amino acid residues and characterized the nanopore translocation of the resultant conjugate strands (Fig. 4, S6, S7, S8, S9†). The current profiles of the ssDNA-peptide conjugates showed a similar pattern as the control described earlier, composed sequentially of an initial steep increase to ∼0.4–0.5 I_open_ depending on the specific mutations, a moderate further increase to ∼0.5–0.6 I_open_, and a subsequent drop to ∼0.4–0.5 I_open_. As a result, we could not determine which neutral/hydrophobic amino acid replaced S_5_ simply by the profile of the peptide-induced current signal. This result might have stemmed from the fact that the constriction of MspA-M2 is not sufficiently narrow to allow accurate discrimination of different amino acids solely based on their molecular contours. We also found MspA-M2 to be able to accommodate the simultaneous passage of a ssDNA and a peptide strand (Fig. S12†).

On the other hand, more pronounced changes were observed when S_5_ was mutated to a negatively charged amino acid residues such as an aspartate or glutamate. The current profiles of both mutants showed a ”small hump” when the mutant amino acid traveled through the constriction, after which the signal slightly attenuated before reverting back to the original current profile(Fig. 4c, d). We then replaced S_5_ with double aspartates or glutamates (designated as S_5_DD and S_5_EE) to ascertain whether this would lead to further signal augmentation. As seen in Fig. 4e and f, the current profiles of both S_5_DD and S_5_EE exhibited an even more pronounced hump that now exceeded the aforementioned peak amplitude levels that we observed in S_5_D and S_5_E. In addition, the hump in the current of mutant G_9_E, in which G_9_ was replaced with glutamate, occurred later than its counterpart in S_5_E or S_5_D (Fig. 4h). These data suggested that the sequential way that the amino acid residues passed through the nanopore constriction played a key role in shaping the signal hump associated with the negatively charged aspartate or glutamate.^13^

Previous studies have revealed, rather counter-intuitively, that nucleotides C and T, although smaller in size than A and G, were more effective in blocking the MspA-M2 nanopore.^28–31^ For example, despite being smaller than poly-A, poly-T generated a lower ionic current when translocating through MspA-M2. Molecular dynamics simulations suggested that thymine had a greater tendency to stack inside the nanopore channel than adenine due to its smaller aromatic rings, which restricted the traverse of ions. Based on the same principle, we hypothesized that the blocking current of a peptide might also be derived mainly from the curving and folding of its backbone chain. Therefore, the observation that negatively charged amino acid residues generated greater ionic currents could be because they were driven downward in the electrical field, which stretched the peptide chain and reduced its thermal motion.^36^ This, in turn, de-blocked the constriction and allowed the passage of more ions.

Because a phosphorylation site carries two negative charges under physiological conditions, we reasoned that its translocation through MspA-M2 should produce a similar change in ionic current as S_5_DD and S_5_EE. Indeed, the current of the polypeptide (GGGGSGGGGSSGGGGSSGGSGGC) phosphorylated at S_5_ began with a steep rising, followed by an extended, high-rising hump, whose amplitude level exceeded those of S_5_E and S_5_D, and was on par with those of S_5_DD and S_5_EE (Fig. 4l). Meanwhile, for the mutant with a phosphorylated G_9_(pS), the signal hump moved to the middle of the peptide-induced signal region (Fig. 4m). Taken together, the data lent credence to the utility of our method in distinguishing between different phosphorylation sites, which could potentially extend to other post-translational modifications.

**Fig. 5:**
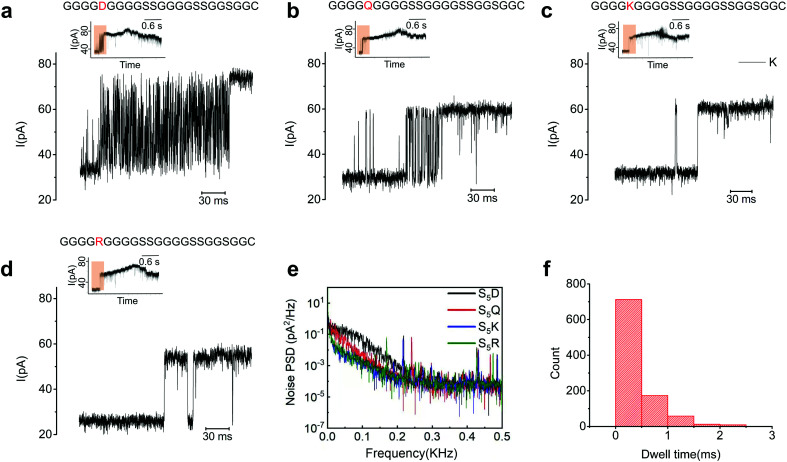
The ionic current profile at the transition region. (a) S_5_D and (b) S_5_Q showing intense upward spikes, compared to S_5_K (c) and S_5_R (d) with few upward spikes. The signal transition region is marked by brown in the inserted figures. (e) Characterization of the noise for S_5_D (black), S_5_Q (red), S_5_K (blue), and S_5_R (green). Below 300 Hz, the noise power spectral density of S_5_D and S_5_Q is higher than S_5_K and S_5_R, contributed mainly by the upward spikes with frequency characteristics ranging from tens to a few hundred hertz. (f) Statistics showing that the dwell time of the upward spikes in (a) is in the range of a few milliseconds, which is much shorter than that of the helicase-induced step movement.

A careful examination of the blocking current at the ssDNA-peptide transition zone revealed a dense array of upward spikes for all the peptide mutants we tested, except for S_5_R and S_5_K (Fig. 5a–e). The average dwell time of each spike was shorter than 1 ms, far below the typical duration of MTA-h-induced step motion (Fig. 5f). We reasoned that these spikes were generated by the mechanical oscillation of the ssDNA-peptide strand in resemblance to the movement of a strained spring upon release. The electrical field distribution can be roughly estimated by *E* = *j*/*σ*, where *E* is the electric field, *j* is the ionic current density, and *s* is the conductivity. Based on the equation, the electrical field is the strongest at the constriction region, where the ionic current density is the highest.^31^ During the translocation of the conjugate strands, the ssDNA entered the pore first and behaved largely as a stretched spring due to the collective modulation of MTA-h and the electrical field. As the peptide segment, which carried much fewer net charges, approached the constriction, the electrostatic force attenuated, which, similar to the release of a strained spring, caused the ssDNA to oscillate. The oscillation dragged part of the peptide in and out of the nanopore in rapid succession, manifested in upward current spikes. In the case of S_5_R and S_5_K, the electrical field exerted an upward force on the positively charged amino acid residues, which could accelerate the peptide translocation and thus mitigate the oscillation. The absence of upward spikes was also observed when G_9_ was replaced by R, but not when a position too far away from the peptide N-terminus was mutated (R_19_), possibly due to the weakening of the electrical field (Fig. S13†). These findings suggested that the ionic current generated by peptide translocation is governed by a complex set of mechanisms. Further studies, including more detailed molecular dynamics simulations, are needed to elucidate the transition states of various amino acid residues inside the MspA-M2 nanopore.

## Conclusion

Here, we have developed a strategy to regulate peptide translocation through the MspA-M2 nanopore and demonstrated its utility in detecting single amino acid residues. The fact that the constriction of MspA-M2 is located at the very bottom of its pore channel allows a read length of up to 17 amino acid residues. The nanopore system that we established could distinguish between amino acid residues with different charge states, and enabled the analysis of post-translational modifications such as serine phosphorylation. We also demonstrated that by using a dual-helicase system, a single peptide could be repeatedly sequenced, potentially leading to significant improvement in sequencing accuracy. Unfortunately, unlike nanopore-based DNA sequencing, peptide translocation did not generate a clear and reproducible step-like ionic current in this study, which precluded sequential discrimination of individual amino acid residues. This observation might be due to the smoothing effect of thermal motion on the blocking current generated by different amino acid residues in an unstrained peptide. Notably, there have recently been studies that achieved step-like blocking current by applying a similar strategy to peptides with a high percentage of negatively charged amino acid residues, confirming the importance of stretching the peptide inside the nanopore.^37,38^ Potential solutions include engineering MspA-M2 to improve the spatial discrimination of its constriction zone, and stretching the peptide chain by applying electro-osmotic flow or *via* an enzymatic approach. These improvements could pave the way for our strategy to eventually achieve high-throughput, low-cost single molecule peptide sequencing, with enormous potential in scientific discovery and clinical applications.

## Data availability

Further data are accessible upon request.

## Author contribution

J. B. was responsible for the overall research design. Z. C. and Z. W. conducted all the experiments for the study, with input from Y. X., Z. C., Z. W., J. B. analysed experimental data, with input from X. Z and B. T., Z. C. and Z. W. generated all figures used in this study. J. B., Z. C. and Z. W. prepared the manuscript. All authors discussed the results and contributed to the final manuscript.

## Conflicts of interest

A patent application has been filed by Tsinghua University, which contains the technologies described in this study, with J. B. and Z. C. listed as the inventors.

## Supplementary Material

SC-012-D1SC04342K-s001

## References

[cit1] Walsh K. A., Ericsson L. H., Parmelee D. C., Titani K. (1981). Annu. Rev. Biochem..

[cit2] Restrepo-Pérez L., Joo C., Dekker C. (2018). Nat. Nanotechnol..

[cit3] Bradley C. V., Williams D. H., Hanley M. R. (1982). Biochem. Biophys. Res. Commun..

[cit4] Zhong H., Zhang Y., Wen Z., Li L. (2004). Nat. Biotechnol..

[cit5] Miyashita M. (2001). Proc. Natl. Acad. Sci. U. S. A..

[cit6] Yates J. R. (2011). Nat. Methods.

[cit7] Yao Y., Docter M., Ginkel J., Ridder D., Joo C. (2015). Phys. Biol..

[cit8] Swaminathan J., Boulgakov A. A., Hernandez E. T., Bardo A. M., Bachman J. L., Marotta J., Johnson A. M., Anslyn E. V., Marcotte E. M. (2018). Nat. Biotechnol..

[cit9] Venkatesan B. M., Bashir R. (2011). Nat. Nanotechnol..

[cit10] Waduge P. (2017). ACS Nano.

[cit11] Pastoriza-Gallego M. (2011). J. Am. Chem. Soc..

[cit12] Piguet F., Ouldali H., Pastoriza-Gallego M., Manivet P., Pelta J., Oukhaled A. (2018). Nat. Commun..

[cit13] Ouldali H., Sarthak K., Ensslen T., Piguet F., Manivet P., Pelta J., Behrends J. C., Aksimentiev A., Oukhaled A. (2020). Nat. Biotechnol..

[cit14] Talaga D. S., Li J. (2009). J. Am. Chem. Soc..

[cit15] Restrepo-Pérez L., John S., Aksimentiev A., Joo C., Dekker C. (2017). Nanoscale.

[cit16] Nivala J., Marks D. B., Akeson M. (2013). Nat. Biotechnol..

[cit17] Nivala J., Mulroney L., Li G., Schreiber J., Akeson M. (2014). ACS Nano.

[cit18] LaBreck C. J., May S., Viola M. G., Conti J., Camberg J. L. (2017). Front. Mol. Biosci..

[cit19] Whitman J. C., Paw B. H., Chung J. (2018). Hematol. Transfus. Cell Ther..

[cit20] Derrington I. M., Craig J. M., Stava E., Laszlo A. H., Ross B. C., Brinkerhoff H., Nova I. C., Doering K., Tickman B. I., Ronaghi M., Mandell J. G., Gunderson K. L., Gundlach J. H. (2015). Nat. Biotechnol..

[cit21] Yan S., Li X., Zhang P., Wang Y., Chen H. Y., Huang S., Yu H. (2019). Chem. Sci..

[cit22] Zhang J., Yan S., Chang L., Guo W., Wang Y., Wang Y., Zhang P., Chen H. Y., Huang S. (2020). iScience.

[cit23] Carter J. M., Hussain S. (2017). Wellcome Open Res..

[cit24] Zhou W., Qiu H., Guo Y., Guo W. (2020). J. Phys. Chem. B.

[cit25] Bonome E. L., Cecconi F., Chinappi M. (2017). Microfluid. Nanofluid..

[cit26] Boukhet M., Piguet F., Ouldali H., Pastoriza-Gallego M., Pelta J., Oukhaled A. (2016). Nanoscale.

[cit27] Manrao E. A. (2012). Nat. Biotechnol..

[cit28] Laszlo A. H., Derrington I. M., Ross B. C., Brinkerhoff H., Adey A., Nova I. C., Craig J. M., Langford K. W., Samson J. M., Daza R., Doering K., Shendure J., Gundlach J. H. (2014). Nat. Biotechnol..

[cit29] Laszlo A. H., Derrington I. M., Ross B. C., Brinkerhoff H., Adey A., Nova I. C., Craig J. M., Langford K. W., Samson J. M., Daza R., Doering K., Shendure J., Gundlach J. H. (2014). Nat. Biotechnol..

[cit30] Bhattacharya S., Derrington I. M., Pavlenok M., Niederweis M., Gundlach J. H., Aksimentiev A. (2012). ACS Nano.

[cit31] Bhattacharya S., Yoo J., Aksimentiev A. (2016). ACS Nano.

[cit32] Bhagavan N. V. and Ha C.-E., in Essentials of Medical Biochemistry, ed. N. V. Bhagavan and C.-E. Ha, Academic Press, San Diego, 2015, 2nd edn, pp. 31–51, doi: 10.1016/B978-0-12-416687-5.00004-X

[cit33] Faller M., Niederweis M., Schulz G. E. (2004). Science.

[cit34] Travers K. J., Chin C. S., Rank D. R., Eid J. S., Turner S. W. (2010). Nucleic Acids Res..

[cit35] Rhoads A., Au K. F. (2015). Genomics, Proteomics Bioinf..

[cit36] Di Muccio G., Rossini A. E., Di Marino D., Zollo G., Chinappi M. (2019). Sci. Rep..

[cit37] Yan S., Zhang J., Wang Y., Guo W., Zhang S., Liu Y., Cao J., Wang Y., Wang L., Ma F., Zhang P., Chen H.-Y., Huang S. (2021). Nano Lett..

[cit38] Brinkerhoff H., Kang A. S. W., Liu J., Aksimentiev A., Dekker C. (2021). Science.

