# Supplementary material for: Controlled movement of ssDNA conjugated peptide through Mycobacterium smegmatis porin A (MspA) nanopore by a helicase motor for peptide sequencing application
Source: Chem Sci. 2021 Nov 12;12(47):15750–6. doi: 10.1039/d1sc04342k (PMC8654031; doi:10.1039/d1sc04342k)
Supplement: SC-012-D1SC04342K-s001 [file SC-012-D1SC04342K-s001.pdf]

Electronic Supplementary Material (ESI) for Chemical Science.
This journal is © The Royal Society of Chemistry 2021

Supporting Information

Controlled movement of ssDNA conjugated peptide through MspA-M2 nanopore by a helicase motor for peptide sequencing application

Zhijie Chen[‡a], Zhenqin Wang[‡a], Yang Xu[a], Xiaochun Zhang[a], Boxue Tian[a], Jingwei Bai[□a]

[a.] School of Pharmaceutical Sciences, Tsinghua University, 100084, Beijing, China;

[‡] These authors contributed equally to this work.

* Corresponding author. Email: jingwbai@tsinghua.edu.cn.

Experimental method

MspA-M2 expression and purification. Gene of MspA-M2 nanopore protein was synthesized and constructed to pET-28b expression vector without additional purification tag.[1, 2] For transformation, 120 ng of pET28b-MspA-M2 plasmid was transferred into 100 μL of E.coli competent cells BL21 (DE3), placed on ice for 30 min, then heated shock at 42 °C for 1.5 min. After that, the cell solution was added with 200 μL of LB liquid medium (LB), and incubated at 37 °C for 60 min. The cell solution was spread on LB agar and placed in a 37 °C incubator for 14 hours. A single colony was picked to 12 mL of LB medium containing 0.6 mg of kanamycin sulfate and shaken at 37 ℃ 220 rpm for 8-12 hours. We then transferred 5 mL of cell solution to 1 L LB containing 50 mg kanamycin sulfate. After growth to OD of 0.6-0.8, 0.5 mM IPTG was added to induce the protein expression overnight. Then, the cells were harvest by centrifuge and resuspended in buffer (20 mM Tris-HCl, 100 mM NaCl, 0.5% (v/v) OPOE, 0.5 mM EDTA, 1 mM DTT, pH 8.0). The cell was then lysed by high-pressure homogenization, and then cooked in water bath at 60 °C for more than 45 minutes. After centrifugation at 18000 g for 30 min, double volume of saturated ammonium sulfate was added to precipitate the protein overnight at 4 °C. The MspA protein was pelleted after centrifugation at 20000 g for 25 min. The pellets were then resuspended in 20-30 mL Buffer (20 mM Tris-HCl and 0.5% (v/v) OPOE, pH 8.0), loaded to Q-column and eluted with salt gradient. After verification by SDS-PAGE, we collected the portions of elution containing the target protein and dialyzed it in buffer (20 mM Tris-HCl, 200 mM NaCl, 0.5% OPOE, pH 8.0). The dialysate was concentrated to 2 mL with a 100 kDa ultrafiltration tube, and loaded into SD200 size exclusion column to obtain high-purity protein **(Fig. S1a)**

2. **MTA helicase expression and purification.** The gene of monomer MTA helicase is provided

by Qitan Technology.[3] In the case of helicase dimer, we connect two MTA helicase monomers head -to-tail with a linker of (AHIVMVDAYKPT). The gene was constructed to pET-15b vector with 7 consecutive histidine on the N-terminal of protein sequence to facilitate the purification.

The transformation and expression of the helicase protein, both monomer and dimer, were the same as the above MspA protein except the use of antibiotics. After expression, the bacteria cell pellets were resuspended in Buffer (50 mM Tris-HCl, 500 mM NaCl, 1 mM DTT, 1 mM EDTA, 5%glycerol, pH 8.0), and lysed with high pressure homogenizer. After centrifuge, the supernatant was added with 60% ammonium sulfate and incubated at 4 °C for 2 hours. The salting-out solution was centrifuged at 18000 g for 30 min, and the precipitated pallets were dissolved in 20-30 mL of buffer (50 mM Tris-HCl, 1 mM DTT, 1 mM EDTA, 5%glycerol,pH 8.0). The solution was loaded on the Ni-NTA column, washed with 15 mM imidazole and eluted with 250 mM imidazole. Then, the sample was dialysis and further purified with Heparin column and SD 200 size exclusion column. The purified sample was verified by SDS-PAGE (Fig. S1b).

3. Verify the MTA helicase unzipping activity and binding strength with ssDNA. The activity of MTA helicase was verified by monitoring the unzipping of targeted double stranded DNA which generate fluorescent signals upon two single-strands dissociation. As shown in **Fig. S2a**, oligo b is labeled with FAM fluorescent molecule at the 5' end and it is quenched by the BHQ-1 molecule which is labeled on the 3' end of oligo d. The helicase induced unzipping event separated the two strands so that the FAM can emit fluorescent signal. Oligo f in the solution can hybridize with released oligo d, thus retaining fluorescent levels by preventing the re-annealing of b and d strand. Briefly, 100 nM of pre-annealed b+d dsDNA and 1 μM oligo f **(Table S1)** were mixed in solution (5 mM HEPES, 5 mM ATP, 5 mM $MgCl_2$, and KCl with different concentration). Then, MTA helicase monomer or dimer was added to reach final concentration of 100 nM. The fluorescent signal is monitored under 488 nm excitation and 515 nm emission setup (**Fig. S2b**).

The binding affinity of helicase to ssDNA is studied with fluorescent polarization methods.[4] According to literatures, we synthesized the 44 nt oligo g with a Hexachloro fluorescein (HEX) attached to the thymine base at position 37. The MTA helicase monomer or dimer was buffer-exchanged to 50 mM HEPES pH 8.0 solution with different salt concentration. To start the measurement, 0.78 nM to 800 nM helicase was mixed with 1 nM oligo g, and the fluorescent

anisotropy was assessed over the course of 20 min at 25 °C.

4. **Conjugation of oligo peptide with ssDNA.** As a model system, the oligo peptide **Table S3** used in this study was designed and synthesized with N-terminal azide and C-termial cysteine. The handle-ssDNA including 8 different sequences are shown in **Table S2**. In this way, different peptide can be tested together in one nanopore experiment and separated from each other by the handle-ssDNA signal, which can largely improve the sequencing throughput. The handle-ssDNA were synthesized with DBCO group at 5'end, and the lead-ssDNA with poly-T sequence was synthesized with maleimide group at the 3'end **(Fig.S3a)**. The conjugation reaction was conducted in 1x PBS buffer with handle: peptide: lead ratio of 1:2:1. The reaction was shaken at 30 °C for 18 hours. The conjugation product was verified by 8% 7 M urea polyacrylamide denatured gel electrophoresis **(Fig. S3b)**.

5. **Electrophysiology recordings and data analysis.** All electrophysiology measurements were performed according to literature.[5] Briefly, a measurement chamber is consisted of two compartments separated by a plastic tube with an orifice (~25 μm in diameter). Before the measurement, the compartments were first cleaned with isopropanol, H_2O_2 and Milli-Q water, followed by drying with compressed nitrogen gas. A pair of Ag/AgCl electrodes purchased from Warner were inserted in each compartment to conduct current. In principle, the compartment that is electrically grounded is defined as the cis and the opposing side is defined as the trans side. The orifice on the tube was then treated with 1,2-Diphytanoyl-sn-glycero-3-phosphocholine (DPhPC) (Avanti Polar Lipids) in hexane, and leave for drying. Approximately 0.1 mL of electrolyte buffer (10 mM HEPES, 400 mM KCl, 5 mM Mg^{2+}, pH 8.0) was added to both compartments. We use a pipette to form and drag an air bubble across the orifice, which will introduce a self-assembled phospholipid bilayer across the orifice. MspA-M2 nanopores protein were diluted and added to cis chamber to obtain spontaneous pore insertions. The cis chamber was immediately perfused with fresh buffer upon the signal of single-pore insertion was obtained.

To prepare the sequencing solution, the peptide DNA conjugate was firstly annealed with a cholesterol-tagged ssDNA oligo (tetheroligo), and then combined with 500 nM MTA helicase

solution, before added to the cis chamber. Under 180 mV applied voltage, the sequencing is initiated by addition of 2 μL 100 mM ATP (Thermo Fisher) to the cis chamber. The electrophysiology signals were amplified by an Axopatch 200B patch clamp amplifier and digitized by a Digidata 1550B digital-to-analog converter (Molecular Devices). The ionic current signal is sampled with a 25 kHz sampling rate and the output was filtered at a 1 kHz angular frequency. Some of the experiments were performed with a homemade trans-impedance amplifier paired with a digitizer from National Instrument. Except the study of the upward-spike phenomenon, all the raw data was filtered with an additional 8-pole Bessel-filter at 100 Hz before analysis and plotting.

The sequencing experiment was typically performed for 10 minutes to 1 hour, during which the solution evaporation or the temperature changes could affect the nanopore system causes some extent of fluctuation of the current. To compensate the current drifting, we firstly calculated the I_{res}/I_{open} (the ratio between blocking current to the open pore current), and then linear scale the obtained overall I_{res}/I_{open} curve to make sure the value of poly-T signal equal to 0.19, which is the typical value of poly-T in previous studies and in our experiment. These correction factors allowed for proper comparison between levels in most peptide sequencing events.

Table S1 oligo sequence for MTA activity test.

Name	Sequence
Oligo b	gataagacaa atacaaagaa caaacaatcg ggataagaca tttttttttt tttttttttttttttttttt tttttttttt tttttttttt
Oligo d	tgtcttatcccgattgtttgttctttgtatttgtcttatc
Oligo f	gataagacaaatacaaagaacaaacaatcgggataagaca

Table S2 “handle” ssDNA, “lead” ssDNA, cholesterol tag DNA sequence

Name	Sequence
1-T89	TTTTTTTTTCCTTTTTTTTCCTCTACCACTTTTCAgATCTCACTATC<br>gCATTCTCATgCAggTCgTAgCTTTTTTCTTTTTTCATCATC
2-T89	TTTTTTTTTTTTTTTTTTTTTTTTTTTTTTTTTCAgATCTCACTATCg<br>CATTCTCATgCAggTCgTAgCTTTTTTCTTTTTTCATCATC
3-T89	TTTTTTTTTTTTACGAGTTTTTTTTTTTTTTTTCAgATCTCACTATC<br>gCATTCTCATgCAggTCgTAgCTTTTTTCTTTTTTCATCATC
4-T89	TTTTTTTTTACTTTTTTTTTTCCTTTTTTTTTTCAgATCTCACTATC<br>gCATTCTCATgCAggTCgTAgCTTTTTTCTTTTTTCATCATC
5-T89	TTTTTTTTTTTACGAGTTTTTTTTTTAATTTTTTTTTTTTTACGAGC<br>gCATTCTCATgCAggTCgTAgCTTTTTTCTTTTTTCATCATC
6-T89	TTTTTTTTTCCTTTTTTTACTTTTTTTTCCTTTTTTTTCTCACTATC<br>gCATTCTCATgCAggTCgTAgCTTTTTTCTTTTTTCATCATC
7-T89	TTTTTTTTTCTTTTTTTAATTTTTTTACTTTTTTTCCTTTTTTTACCg<br>CATTCTCATgCAggTCgTAgCTTTTTTCTTTTTTCATCATC
8-T89	TTTTTTTTTTTGTTTTTTTTCTCATCTGTTTTTTTTTTCTCACTATC<br>gCATTCTCATgCAggTCgTAgCTTTTTTCTTTTTTCATCATC
Lead- ssDNA	TTTTTTTTTTTTTTTTTTTTTTTTTTTTTT
Tether oligo	TTTTTTTTTTTTTTTgCTACgACCTgCATgAgAATTTTTTTTTTTTT<br>TTTTTTTTTTTTTTTTTTTTTTTTTTTT

Table S3 peptide sequence

5th mutation	5th and 6th mutation
GGGGDGGGGSSGGGGSSGGSGGC	GGGGDDGGGGSSGGGGSSGGSGGC
GGGGEGGGGSSGGGGSSGGSGGC	GGGGEEGGGGSSGGGGSSGGSGGC
GGGGKGGGGSSGGGGSSGGSGGC	GGGGKKGGGGSSGGGGSSGGSGGC
GGGGQGGGGSSGGGGSSGGSGGC	GGGGQQGGGGSSGGGGSSGGSGGC
GGGGWGGGGSSGGGGSSGGSGGC	GGGGSSGGGGSSGGGGSSGGSGGC
GGGGSGGGGSSGGGGSSGGSGGC	GGGGPPGGGGSSGGGGSSGGSGGC
GGGGNGGGGSSGGGGSSGGSGGC	GGGGNNGGGGSSGGGGSSGGSGGC
GGGGGGGGGSSGGGGSSGGSGGC	GGGGWWGGGGSSGGGGSSGGSGGC
GGGGAGGGGSSGGGGSSGGSGGC	GGGGMMGGGGSSGGGGSSGGSGGC
GGGGVGGGGSSGGGGSSGGSGGC	GGGGLLGGGGSSGGGGSSGGSGGC
GGGGRGGGGSSGGGGSSGGSGGC	GGGGTTGGGGSSGGGGSSGGSGGC
GGGGHGGGGSSGGGGSSGGSGGC	GGGGFFGGGGSSGGGGSSGGSGGC
GGGGPGGGGSSGGGGSSGGSGGC	GGGGHHGGGGSSGGGGSSGGSGGC
GGGGMGGGGSSGGGGSSGGSGGC	GGGGRRGGGGSSGGGGSSGGSGGC
GGGGTGGGGSSGGGGSSGGSGGC	GGGGVVGGGGSSGGGGSSGGSGGC
GGGGYGGGGSSGGGGSSGGSGGC	GGGGYYGGGGSSGGGGSSGGSGGC
GGGGLGGGGSSGGGGSSGGSGGC	GGGGIIGGGGSSGGGGSSGGSGGC
GGGGIGGGGSSGGGGSSGGSGGC	GGGGAAGGGGSSGGGGSSGGSGGC
GGGGFGGGGSSGGGGSSGGSGGC	GGGGGGGGGGSSGGGGSSGGSGGC
GGGGCGGGGSSGGGGSSGGSGGC	GGGGCCGGGGSSGGGGSSGGSGGC
GGGG(pS)GGGGSSGGGGSSGGSGGC	9th mutation
19th mutation	GGGGSSGGSGSSGGGGSSGGSGGC
GGGGSSGGGGSSGGGGSSRC	GGGGSSGGFGSSGGGGSSGGSGGC
	GGGGSSGGRGSSGGGGSSGGSGGC
	GGGGSSGGEGSSGGGGSSGGSGGC
	GGGGSSGG(pS)GSSGGGGSSGGSGGC

Note: pS means Serine is phosphorylated.

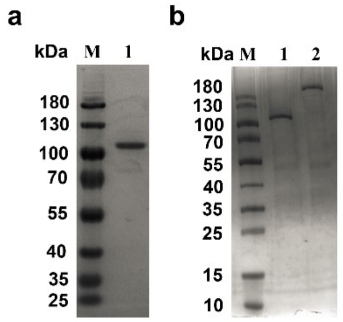

Fig. S1 (a) Gel electrophoresis characterization of purified MspA-M2 nanopore; (b) Purified MTA helicase monomer (lane 1) and dimer (lane 2).

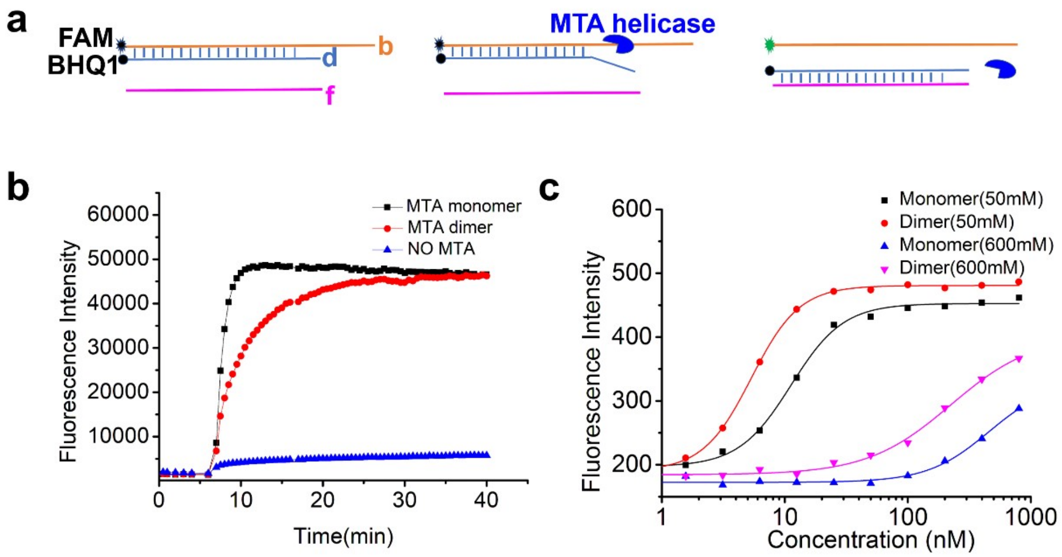

Fig. S2 (a) Fluorescent assay to characterize the helicase unzipping activity. The fluorescence was normally quenched if strand b and d annealed together; when helicase bound and unzipped the double stranded DNA, the BHQ-1 on strand d would be separated away from the FAM molecular on strand b, therefore, the fluorescent signal could be recovered. Strand f was used to bind to strand d upon releasing. Since the helicase required a portion of ssDNA to bind before it can unzip the double stranded portion, the fully compensated d+f dsDNA would not be unzipped by the helicase. Therefore, the existing of strand f can stabilized the fluorescent signal by preventing reannealing of strand d to strand b. (b) The activity of MTA helicase monomer and dimer to unzip dsDNA under 600mM KCl. Strong fluorescent signal was observed for both MTA constructs, indicating the unzipping activity under high salt condition. Indeed, the unzipping process of monomer is obviously faster than the dimer, which we hypothesized that the two units in the dimer might not synchronized well during unzipping, resulted in reduced unzipping speed compared to monomer. (c) Fluorescent polarization to measure the binding affinity for monomer and dimer under different salt conditions, which clearly demonstrated the improved binding affinity for helicase dimer.

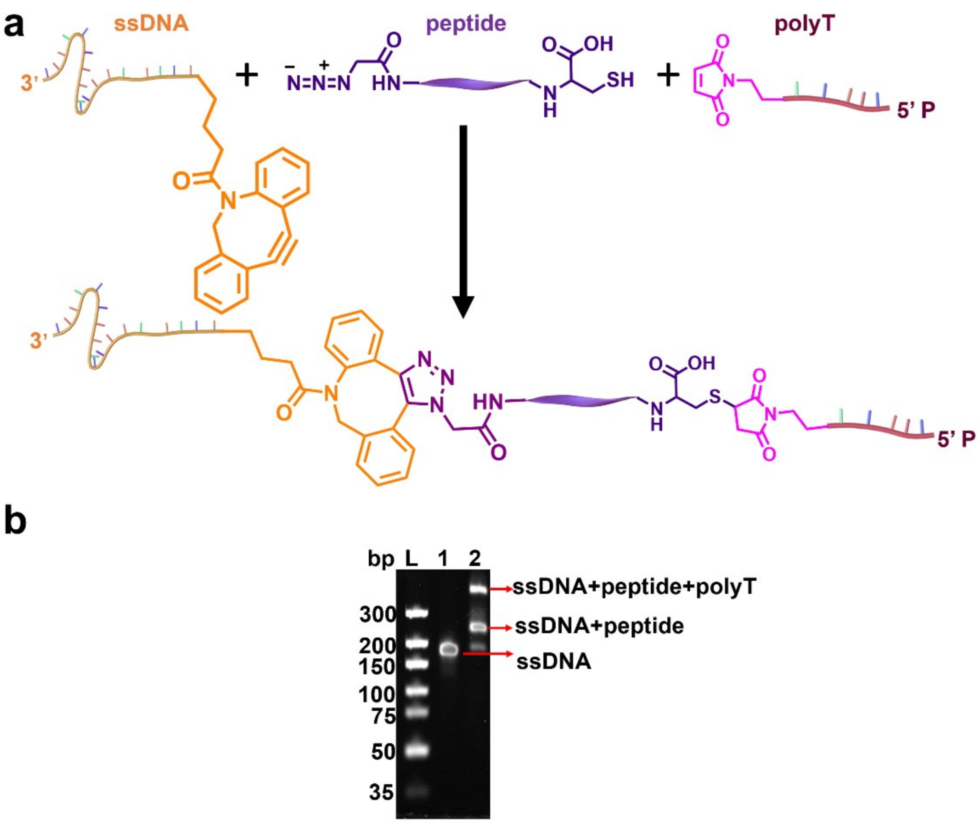

Fig. S3 (a) Schematic of peptide conjugation to handle-ssDNA by azide-DBCO click chemistry, and to poly-T sequence of lead-ssDNA by cysteine-maleimide reaction. (b) Gel electrophoresis characterization after the reaction, showing successfully formation of sandwiched conjugate structure

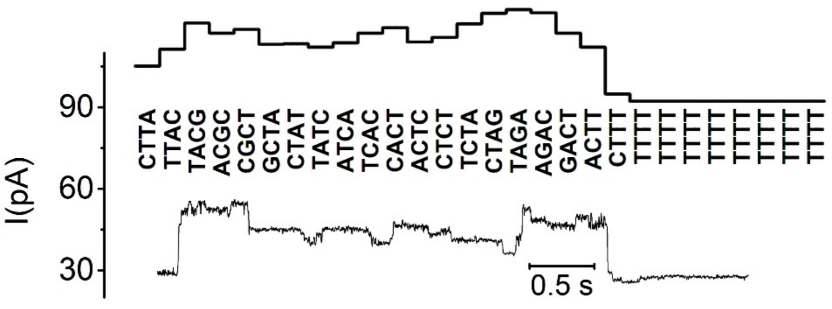

Fig. S4 Ionic current profile of the handle-ssDNA predicted based on the pre-determined quadramer map described in literature (top), and experimentally measured (bottom). The difference may be caused by the salt condition and motor protein in use.

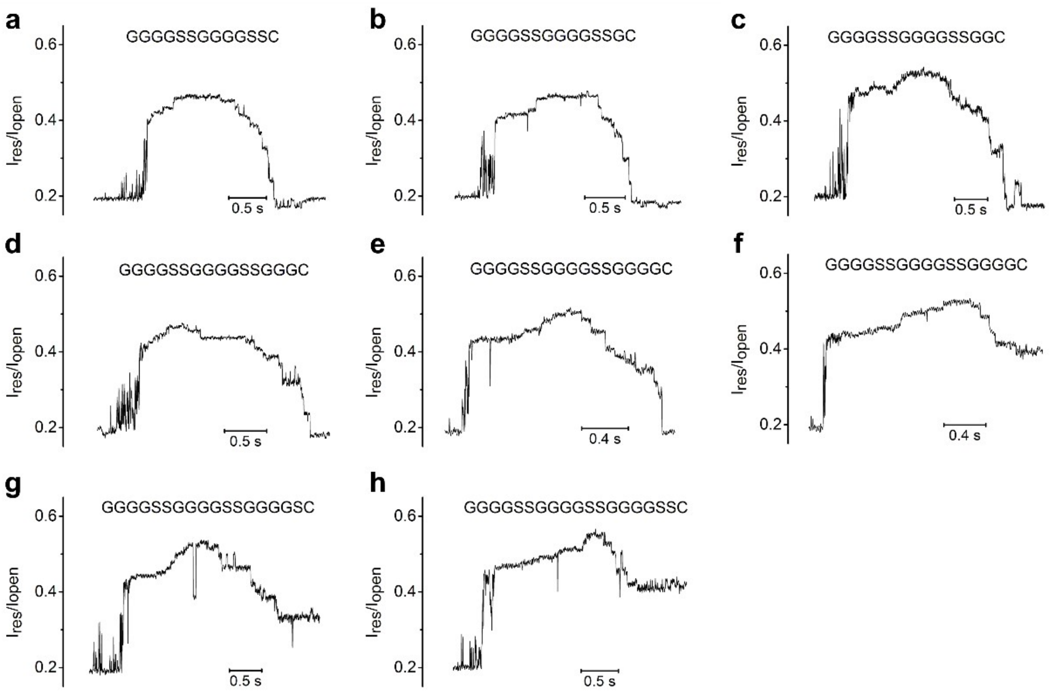

Fig. S5 Representative current trace for non-charged peptide with different lengths. (a)-(d) Peptide length of 13-aa to 16-aa can exhibit poly-T signals from lead-ssDNA. (e) (f) In the case of 17-aa, signal of lead-ssDNA can be detected for about 50% of events. (g) (h) For peptide length of 18-aa and 19-aa, less than 10% of events can detect the poly-T signal contributed by lead-ssDNA.

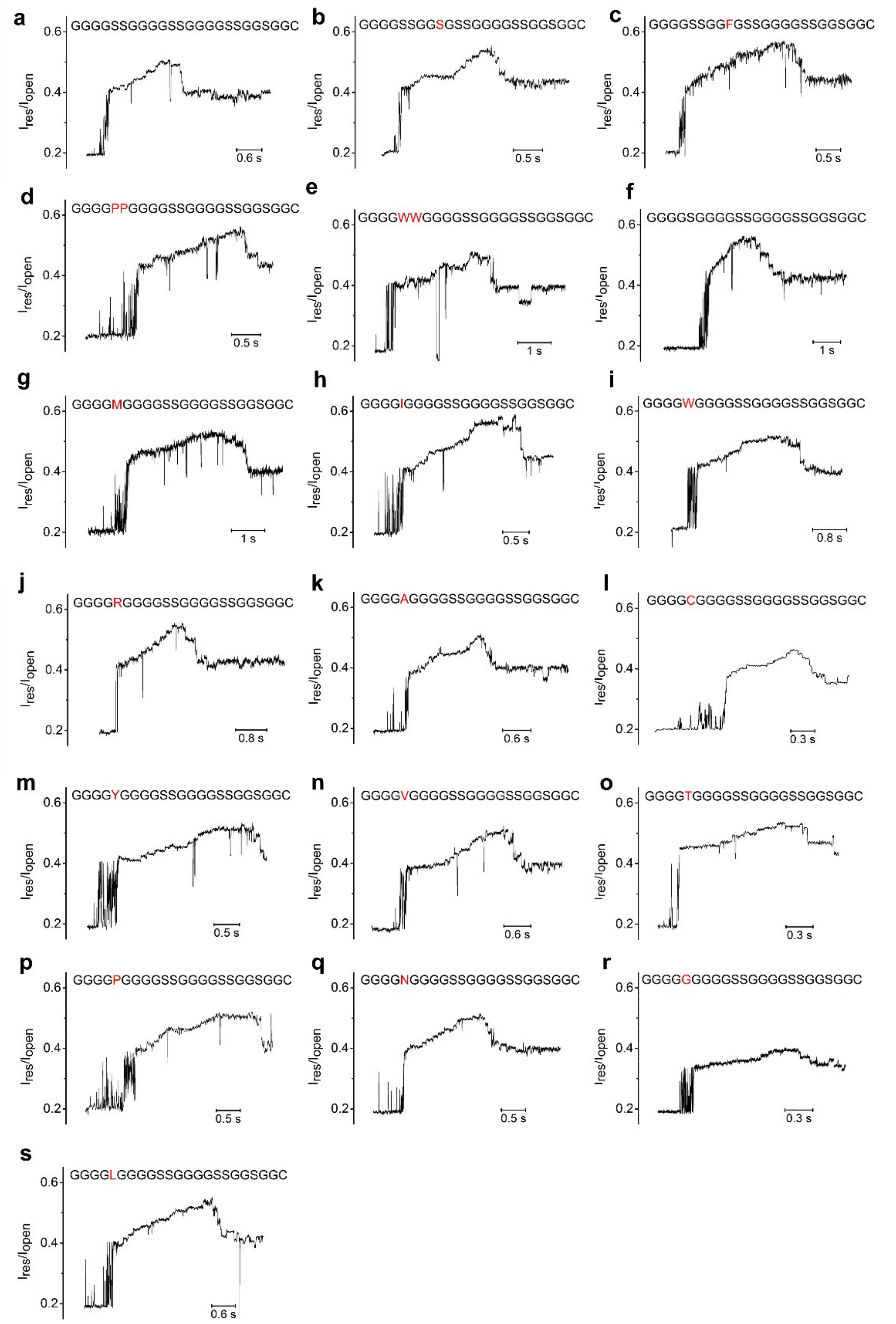

Fig. S6 (a) Current profile for 24-aa peptide with sequence of GGGGSSGGGGSSGGGGSSGGSGGC. Different mutations on the 24-aa with neutral charge amino acid substitutions: (b) G_9S, (c) G_9F, (d) S_5P and S_6P, (e) S_5W and S_6W. (f) Current profile for 23-aa peptide with sequence of GGGGSGGGGSSGGGGSSGGSGGC. Different on the 23-aa with non-negative charge amino acid substitutions: (g) S_5M, (h) S_5I, (i) S_5W, (j) S_5R, (k) S_5A, (l) S_5C, (m) S_5Y, (n) S_5V, (o) S_5T, (p) S_5P, (q) S_5N, (r) S_5G, (s) S_5L

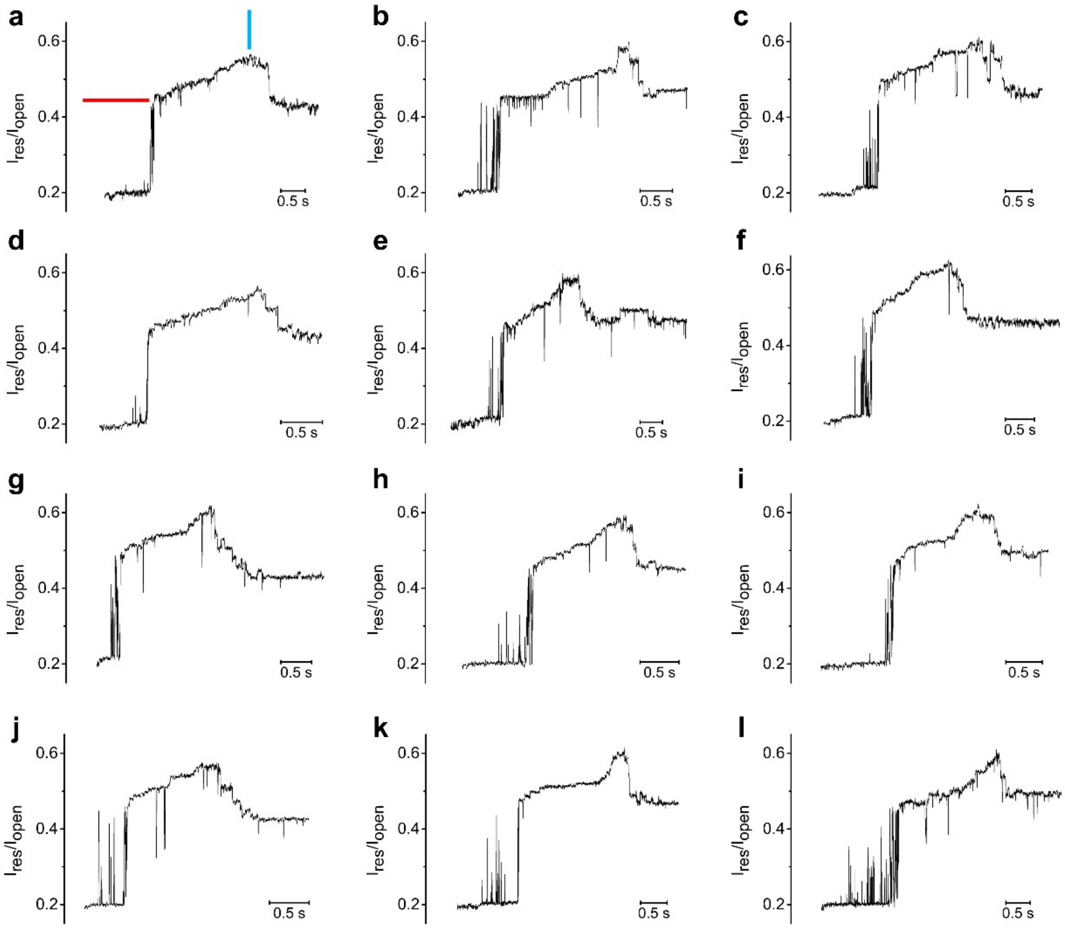

Fig. S7 (a)-(l) Current profile of peptide (GGGGFGGGGSSGGGGSSGGSGGC) to access the reproducibility of the signal. Red and Blue line mark the choice of initial current rising (I_1) and main peak (I_2).

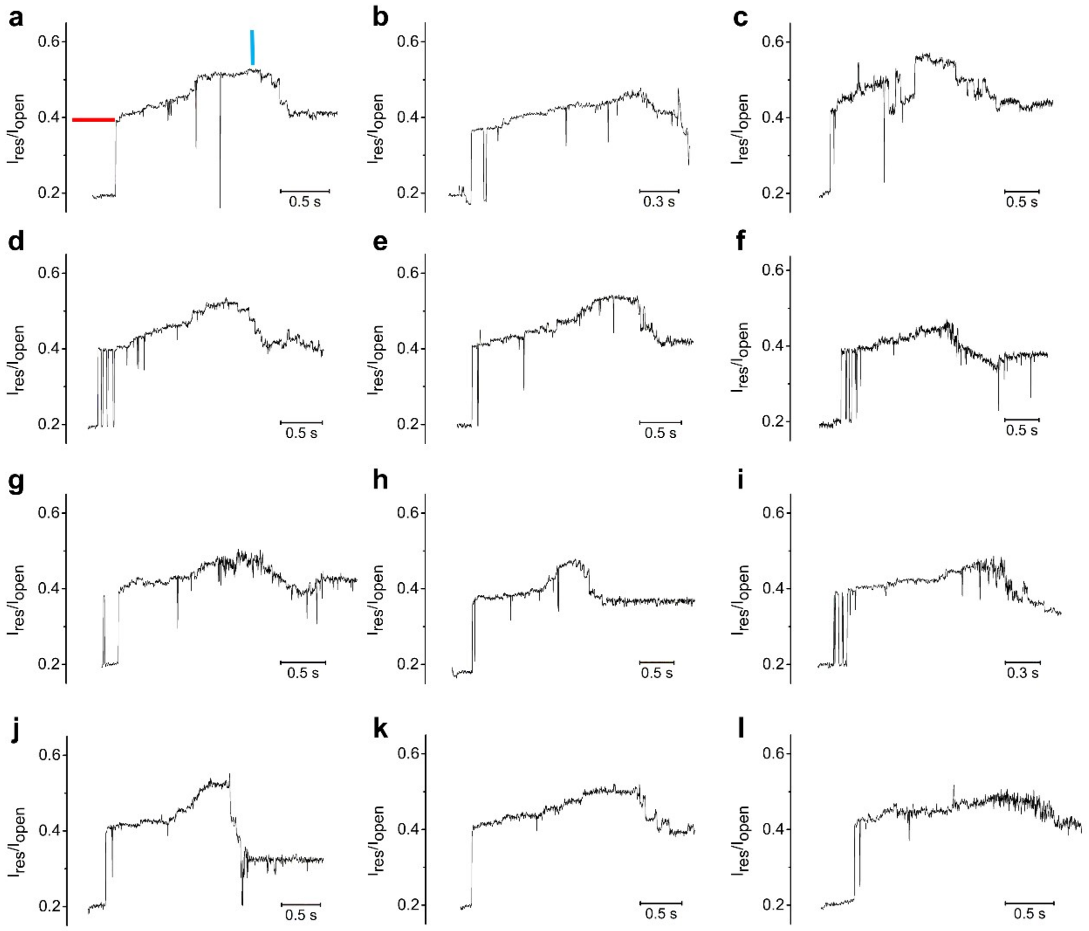

Fig. S8 (a)-(l) Current profile of peptide (GGGGKGGGGSSGGGGSSGGSGGC) to access the reproducibility of the signal. Red and Blue line mark the choice of initial current rising (I_1) and main peak (I_2).

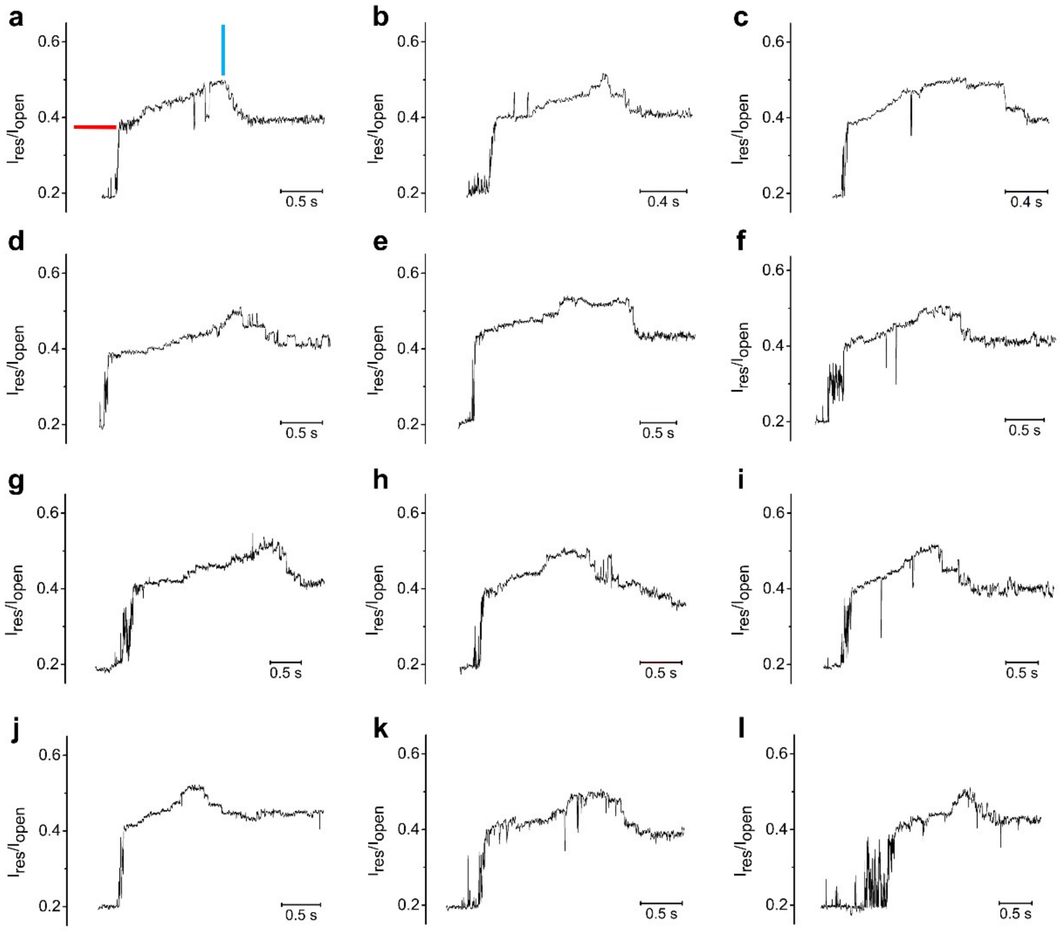

Fig. S9 (a)-(l) Current profile of peptide (GGGGQGGGGSSGGGGSSGGSGGC) to access the reproducibility of the signal. Red and Blue line mark the choice of initial current rising (I_1) and main peak (I_2).

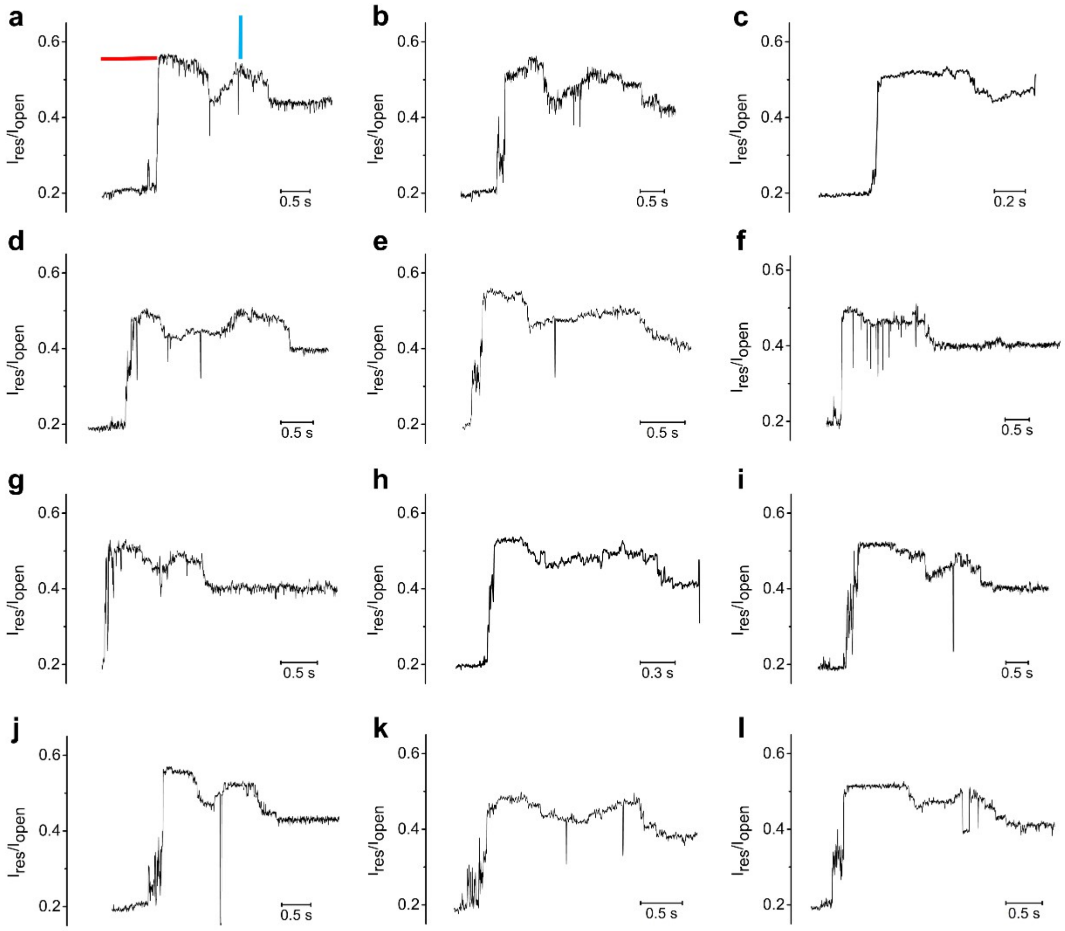

Fig. S10 (a)-(l) Current profile of peptide (GGGGDDGGGGSSGGGGSSGGSGGC) to access the reproducibility of the signal. Red and Blue line mark the choice of initial current rising (I_1) and main peak (I_2).

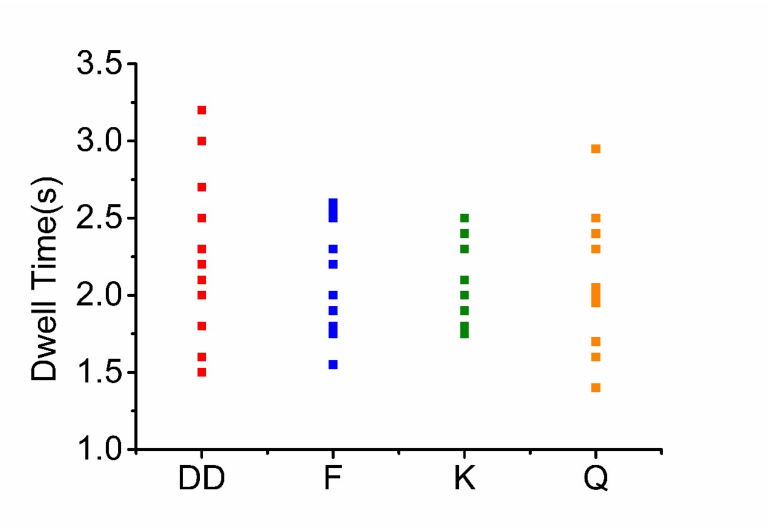

Fig. S11 Effect of point mutation on S_5 of peptide (GGGGSGGGGSSGGGGSSGGSGGC) for the peptide translocation duration. Different type of amino acid residue does not affect the duration of the events apparently, which may be in turn mainly governed by the stochastic motion of the motor protein.

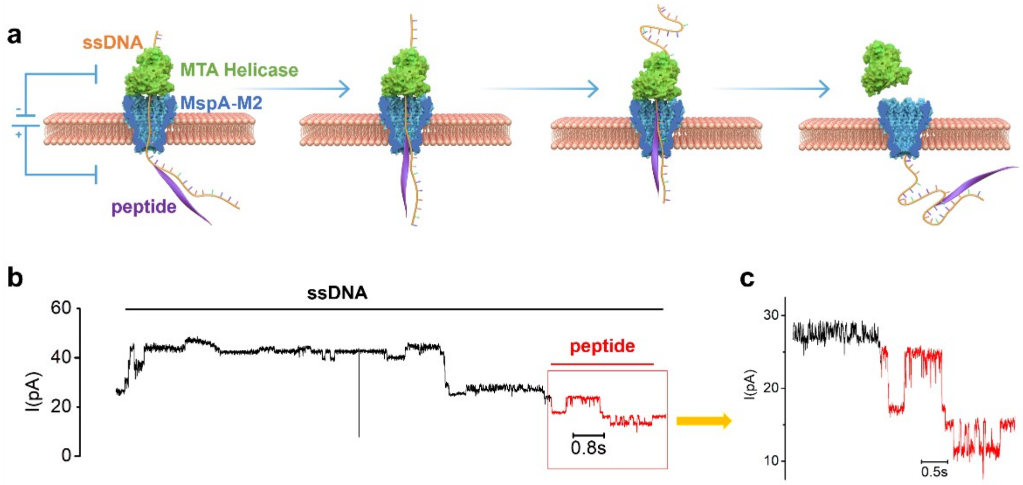

Fig. S12 (a) Schematic of co-translocation of ssDNA-peptide conjugates where the peptide is conjugated as a side chain in the middle of the ssDNA. The peptide under testing is (GGGGSGGGGSSGGGGSSGGSGGC), with N termina linked to the base of T in the 28th nucleotide of ssDNA (TTTTTTTTTTTTTTTTTTTTTTTTTTTTTTTTTTTTCCTCTACCACTTTTCAgATCTCACTATCgCATTCTCATgCAggTCgTAgCTTTTTTCTTTTTTCAT). (b)(c) Experimental result showed that strand of ssDNA and peptide can be pull into the MspA-M2 nanopore at the same time, which showed further decrease of current value with step-like profile. This result showed that the MspA-M2 nanopore may still be a bit too large to provide enough differentiation for amino acids.

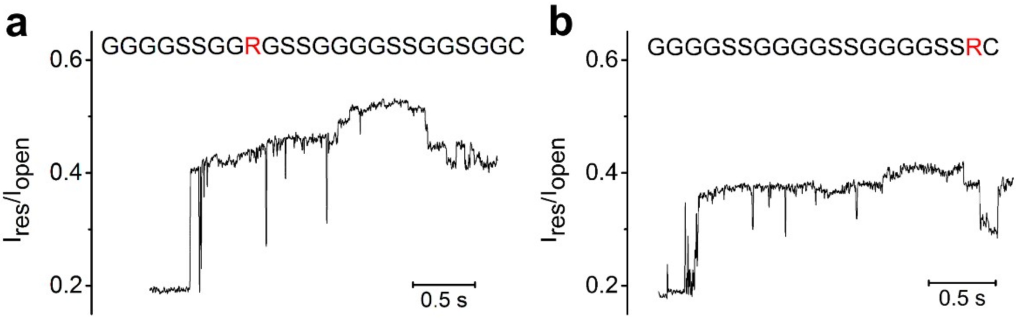

Fig. S13 (a) Current profile of a 23-aa peptide with R_9 marked in the sequence showing almost no detection of upward spike at the ssDNA-to-peptide signal transition region; (b) Current profile of mutation of 20-aa peptide with R_{19} marked in the sequence showing recovery of the upward spike at the ssDNA-to-peptide signal transition region;

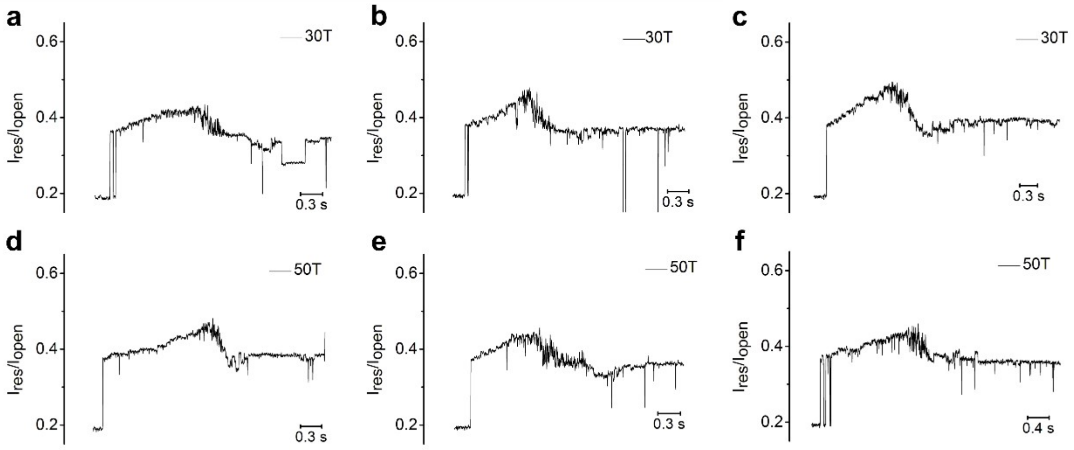

Fig. S14 Current profile of peptide (GGGGKGGGGSSGGGGSSGGSGGC) conjugated with different length of polyT sequence: (a)-(c) 30 nucleotides; (d)-(f) 50 nucleotides, in order to study the effect of polyT sequence on the peptide translocation speed. As a result, no significant changes of translocation time is observed. It is possible that the electrical field out of the *trans* exit quickly attenuated, thus only the nearest nucleotides can contribute to tugging force, so that the length of the poly-T may not affect the overall pulling back process.

References

1. T. Z. Butler, M. Pavlenok, I. Derrington, M. Niederweis and J. Gundlach, *Proceedings of the National Academy of Sciences*, 2008, **105**, 20647 - 20652.
2. C. Heinz, E. Roth and M. Niederweis, *Methods in molecular biology*, 2003, **228**, 139-150.
3. C. Chen, M. Wang, Y. Zhou and T. Fu., Patent application, WO2020061997A1.
4. H. Q. Xu, A. H. Zhang, C. Auclair and X. G. Xi, *Nucleic Acids Res*, 2003, **31**, e70.
5. E. Manrao, I. Derrington, A. H. Laszlo, K. W. Langford, M. K. Hopper, N. Gillgren, M. Pavlenok, M. Niederweis and J. Gundlach, *Nature Biotechnology*, 2012, **30**, 349-353.
